# Cyclic Water Clusters in Tape-Like and Cage-Like Structures

**DOI:** 10.3390/molecules16042871

**Published:** 2011-04-04

**Authors:** Qin Huang, Lihong Diao, Chuang Zhang, Fuhou Lei

**Affiliations:** 1 College of Chemistry and Ecological Engineering, Guangxi University for Nationalities, Nanning 530006, China; 2 Key Laboratory of Development and Application of Forest Chemicals of Guangxi, Nanning 530006, China

**Keywords:** complexes, water clusters, negatively charge, host-guest

## Abstract

Controlling the ratio of 2,2′-bpy to benzene-1,3,5-tricarboxylic acid produces two interesting complexes, namely [Co(2,2′-bpy)_3_]·(SO_4_)·8.5H_2_O (**1**) and [Cu_2_(BTCA) (2,2′-bpy)_4_] (OH)·(2,2′-bpy)_0.5_·14H_2_O (**2**) (H_3_BTCA = benzene-1,3,5-tricarboxylic acid, 2,2′-bpy = 2,2′-bipyridine). We report the structural evidence in the solid state of discrete lamellar water cluster conformations. These units are found to act as supramolecular glue in the aggregation of cobalt (II) or copper (II) complexes to give three dimensional cage-like networks through hydrogen-bonding. It is interesting that the structure of complex **1** contains a 3D negatively charged cage.

## 1. Introduction

There is hardly a compound that has been more thoroughly investigated than water, and this is due to its importance in many biological, chemical, and physical processes [1]. In the past several decades, there has been increasing interest in the experimental and theoretical study of small water clusters [(H_2_O)_n_, where n = 2-16] because these water assemblies are considered to be ideal research models to gain an insight into some of the unexplained properties of bulk water [2], into the processes that occur at the ice-liquid, ice-air, and liquid-air interfaces [3], and into the nature of water-water and water-solute interactions [4,5,6,7,8,9,10,11,12,13,14]. Water clusters can play an important role in the stabilization of supramolecular systems both in solution and in the solid state, and there is clearly a need for a better understanding of how such water aggregations are influenced by the overall structure of their surroundings [15,16,17,18,19,20]. So far, a variety of tetramer [21,22,23], pentamer, hexamer, octamer and decamer water clusters have been isolated in different solid crystalline hosts. Small water clusters are crucial building units for extended water morphologies including tapes [24] and layers [25] whose physical properties are closely associated with those of bulk water. The formation of different water morphologies may be the hydrophilic groups on the host frameworks. Therefore, a systematic variation of these backbones and determination of the corresponding water layers will improve our understanding of the structures of water. In this context, we have succeeded in obtaining two novel cobalt (II) and copper (II) complexes [Co(2,2′-bpy)_3_]·(SO_4_)·8.5H_2_O (**1**) and [Cu_2_(BTCA) (2,2′-bpy)_4_] (OH)·(2,2′-bpy)_0.5_·14H_2_O (**2**) [26,27]. The unusual 3D water cluster is stabilized by the hydrophobic hosts [28,29,30,31,32] that undergo hydrogen-bonding to two adjacent hydrogen bond sheets.

## 2. Results and Discussion

### Crystal Structures of Compounds ***1-2***

**Figure 1:**
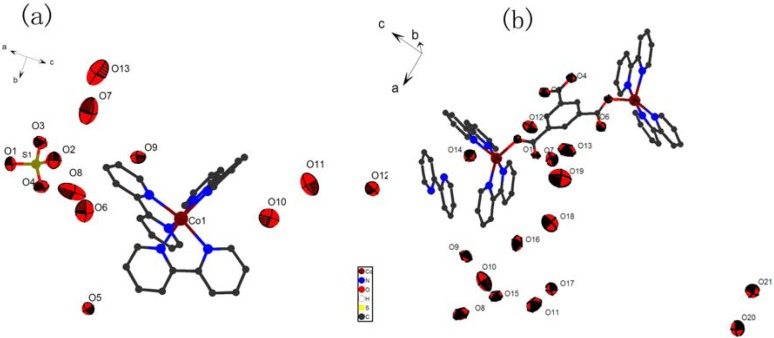
**(a)** The coordination environments of Co atoms with 50% thermal ellipsoids of complex **1**. **(b)** The coordination environments of Cu atoms with 50% thermal ellipsoids of complex **2**.

Single-crystal X-ray diffraction measurement reveals that complex **1** crystallizes in a C2/c space group. A molecular structure showing the arrangement of the Co (II) metal center is shown in Figure 1. The Co atom is six coordinated and adopts distorted octahedral geometry by coordinating to six nitrogen atoms from three 2,2′-bpy molecules (Co(1)-N(1) 2.169(3)Å; Co(1)-N(2) 2.155(3) Å; Co(1)-N(3) 2.165(4) Å; Co(1)-N(4) 2.156(4)Å; Co(1)-N(5) 2.148(3) Å; Co(1)-N(6) 2.179(4) Å) with nearly the same M-N bond distances. One isolated sulfate ion and eight uncoordinated water molecules are contained in this structure.

**Figure 2:**
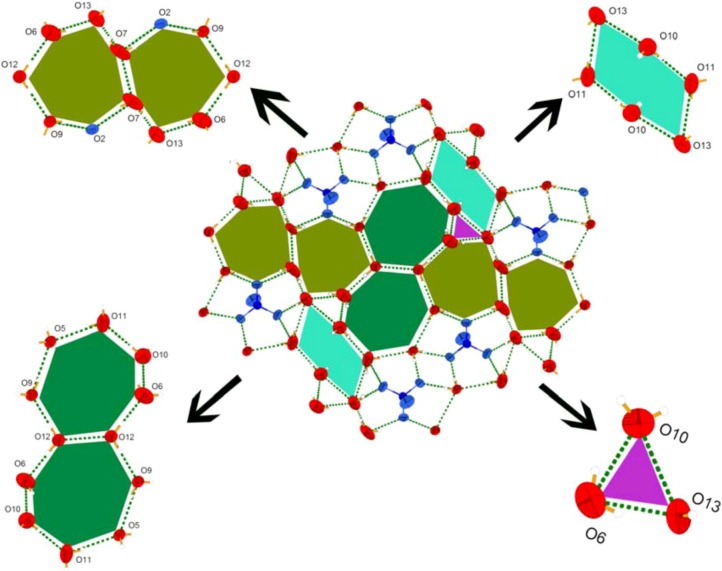
Perspective view of supramolecular water clusters morphology in complex **1**.

**Figure 3:**
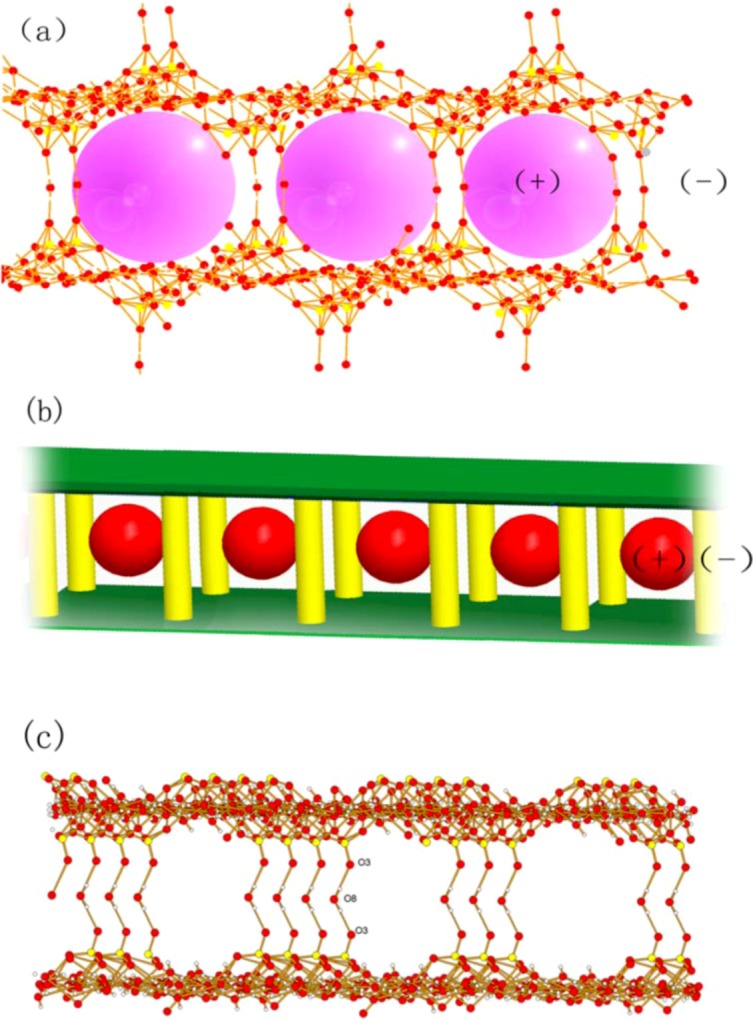
Model of the 3D structure showing the frameworks formed by the guest molecules (pillar-layer structure) with negative charge and the host (red ball) with positive charge.

It must be remarked that the deprotonated sulfate ion and eight lattice water molecules are held together forming hydrogen-bonded layers, as shown in Figure 2. Within the hydrogen-bonded layers, uncoordinated sulfate ion and eight uncoordinated water molecules are linked together via hydrogen-bonding interactions, forming a infinite inorganic-water 2D framework. In this system, the uncoordinated sulfate ion is a host, and acts as a guest of the stacking structure of the title complex (Figure 1). In other organic-water clusters [33,34], the organic compound always acts as a host. Such dual host-guest structure features including water clusters are still very rare. This 2D water layer consists of fused four-, five-, six-, ten- and eighteen-membered rings, as well as corner-sharing four-membered rings (Figure 2). In addition, the structure of the title compound is composed of 2D layers of organic anions [(SO_4_)·13(H_2_O)]^4-^ separated by one water molecule (O8), sulfate ion and dinuclear cobalt complex [Co_2_(2,2'-bpy)_6_]^4+^ pillars resulting in a three-dimensional frameworks (Figure 3c).

It is very interesting that the supramolecular assembly of [(SO_4_)_2_·16(H_2_O)]^4-^ with [Co_2_(2,2′-bpy)_6_]^4+^ forms an negative charged cage (Figure 3). In this cage, the lattice water molecules and sulfate ions accept the hydrogen atoms of the 2,2′-bpy ligand, resulting in a multitude of unconventional interactions. Through the hydrogen bonds, the sulfate ion and lattice water molecules can be linked together to form a negatively charged hydrogen-bonded cage with the identical distance of 12.45Å (Figure 3a and b) recently [35,36].

Complex **2** conforms to the space group C2/c (Table 1). The molecular structure showing the arrangement about the Cu(II) metal center is shown in Figure 1(b). The Cu(II) atom is five coordinated and adopts distorted trigonal biyramid geometry with N6, N8 and O5 (N1, N4 and O2 for Cu2) constituting the base of the square-pyramid, whereas N5 and N7 (N2 and N3 for Cu2) occupy the apical position. (Cu(1)-N(5) 1.970(5) Å; Cu(1)-N(7) 1.992(5) Å; Cu(2)-N(3) 1.980(5) Å; Cu(2)-N(2) 1.987(5) Å). One isolated 2,2′-bpy molecule, one hydroxyl and fourteen uncoordinated water molecules are contained in this structure.

**Figure 4:**
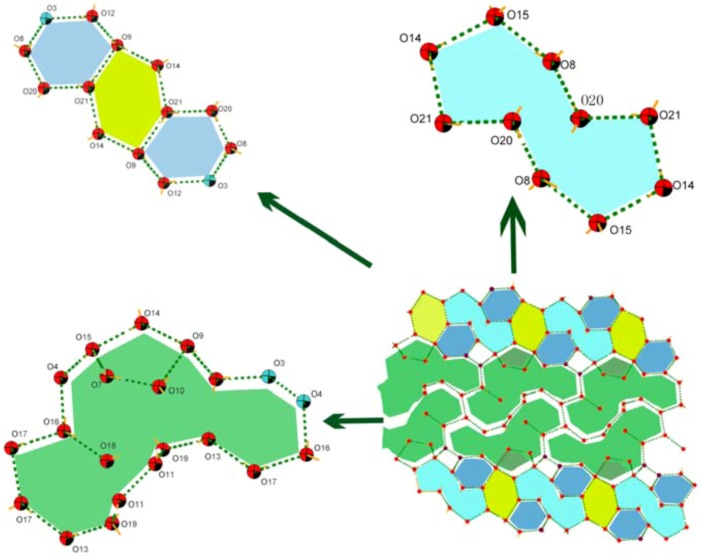
Perspective view of supramolecular water clusters morphology in complex **2**.

**Figure 5:**
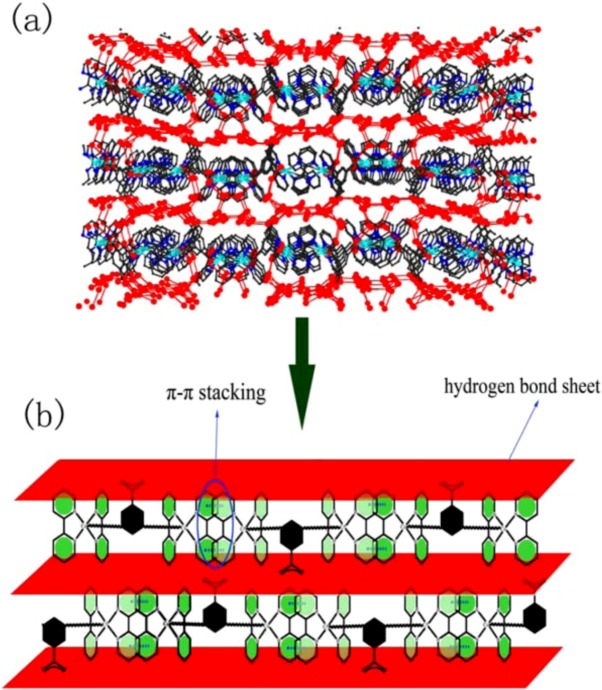
**(a)** The 2D layers of complex 2 along ac plane. **(b)** Perspective view of the water layers, showing the π···π stacking interactions between the 2,2′-bpy molecules of the host.

It must be remarked that sixteen water molecules are connected to each other by hydrogen bonding (Table 2). Furthermore, these water molecules and their symmetric equivalent atoms are joined together forming a 2D water layer (Figure 4). Most of the O···O distances and the O···O···O angles fall around the average of 2.8514Å and 168.074˚, respectively. These values are close to those found in liquid water [37]. These values also indicate that the configuration of the water tape is strongly enforced by the shape of the supporting backbone. This 2D water layer consists of fused four-, five-, six-, ten- and eighteen-membered rings in a ratio of 1:1:3:1:1. Molecular dynamics simulations suggested that liquid water is composed of small cyclic water clusters with different sizes, in which the cyclic water pentamer is the dominant species [38]. The water tapes are separated far from each other (6Å) by the bulging hydrophobic backbone which serves as a pillar (Figure 5). Only weak Van der Waals interactions are found between the bulging areas of the adjacent metal-organic layers.

After inspecting the structure more closely, we found that O7, O8, O11, O15 and O16 are hydrogen-bonded to the carboxyl oxygen atoms (O3 and O4) that are pointing away from the surfaces of the metal-organic framework (O7···O1 = 2.771(8)Å, O8···O3 = 2.692(10) Å, O11···O6 = 2.776(10) Å, O15···O4 = 2.775(8)Å, O16···O4 = 2.762(9) Å). So the benzene-1,3,5-tricarboxylic acid provides potential hydrogen bonding intermolecular interactions, beside its coordination ability towards metal ions. The other oxygen atoms of the lattice water molecules have no significant supramolecular interactions with the metal-organic layers, but they are hydrogen-bonded to each other. Adjacent water rings are assembled in a head-to-head or shoulder-to-shoulder fashion via hydrogen bonds along the ac plane to give an extender water tape occupying the groove region of the surface.

The IR spectral data also confirm the structures. Both the high intensity bands at range of 3,100 to 3,500 cm^-1^ (for complex **1**) and 3,000 to 3,500 cm^-1^ (for complex **2**) were observed, which can be attributed to the vibrations of H_2_O molecules. The vibration of C=O groups are downshifted compared to the free ligand (BTCA) in complex **1** as it coordinated to the metal ions. Medium intensity bands in the 1,556 cm^-1^ (for complex **1**) and 1,590 cm^-1^ region (for complex **2**) were regarded as a combination of C=N of the pyridine and aromatic C=C stretching vibrations.

**Figure 6:**
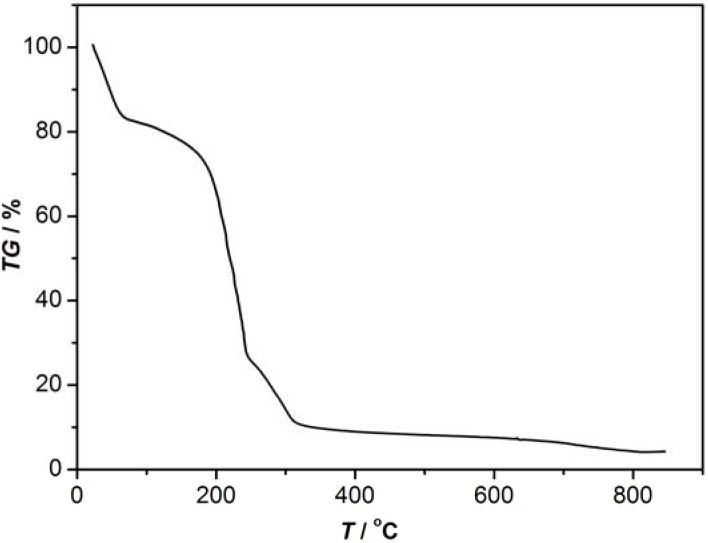
The TG-DTG curve of complex 1.

**Figure 7:**
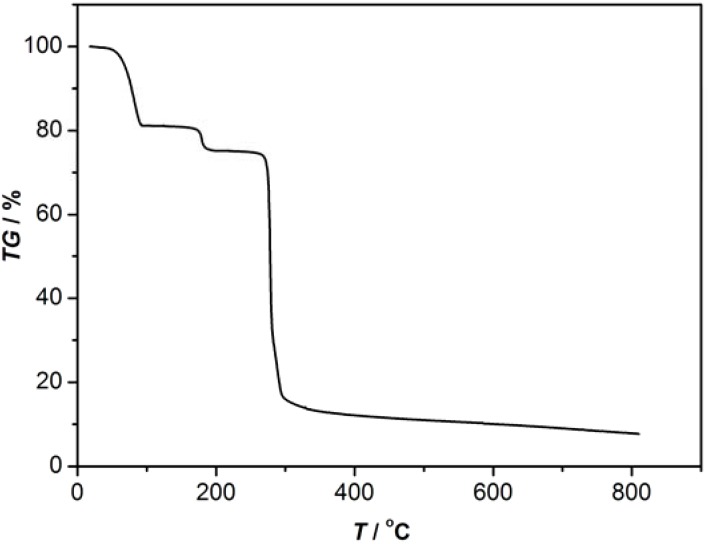
The TG-DTG curve of complex 2.

TG-DTG are carried out in the interest of studying the thermal behavior of complexes **1** and **2**. The TGA indicated that complex **1** loses 19% of its total weight in the 30-80 °C temperature range, which corresponds to the removal of the lattice water molecules (calcd. 19.7%). When the temperature continues to rise, the product loses 55% of the total weight in the 210-230 °C temperature range, corresponding to the removal of the 2,2′-bpy molecules (calcd. 60%) and sulfate ion (Figure 6), cobalt(II) was completely degraded to CoO (observed 9%). Complex **2** displays a similar behavior (Figure 7). The title compound loses 20% of the total weight in the 50-80 °C temperature range, corresponding to the removal of the uncoordinated water molecules. As the temperature rises, the complex loses 5% of the total weight in the 90-160 °C temperature range because of the decomposition of the isolated 2,2′-bpy molecule (calcd. 6%). Then, the compound sheds the coordinated 2,2′-bpy molecules in the 240-250 °C temperature range. The residual percentage weight (9%) at the end of the decomposition of the complex is consistent with the formation of CuO (expected 7%).

**Table 1:** Crystal data and structure refinements for complexes **1-2**.

Complex	**1 **	**2**
Empirical formula	C60H82Co2N12O25S2	C54H49Cu2N9O11
Formula weight	1553.35	1127.11
Temperature(K)	293(2) K	296(2) K
Crystal system	Monoclinic	Monoclinic
Space group	C2/c	C2/c
a(Å)	22.979(4)	12.551(5)
b(Å)	13.656(3)	23.483(9)
c(Å)	24.890(6)	42.330(17)
α(°)	90	90
β(°)	115.235(2)	95.053(6)
γ(°)	90	90
V(Å^3^)	7065(3)	12428(9)
Z	4	8
D_calc_(mgcm^-3^)	1.442	1.397
μ(mm^-1^)	0.614	0.765
F[000]	3204	5456
θ(°)	1.78 to 25.01	0.97 to 25.01
Data/restraints/parameters	6201/ 0 / 457	10944 / 0 / 784
Goodness-of-fit on F^2^	1.020	1.045
Fianl R^a^ indices[I>2σ(I)]	R1 = 0.0609, wR2 = 0.1764	R1 = 0.0746, wR2 = 0.1983
R indices (all data)	R1 = 0.0724, wR2 = 0.1900	R1 = 0.1118, wR2 = 0.2244

**Table 2:** H-bonded lengths (Å) and angles (˚) of complexes **1** and **2**.

D-H···A	d(H···A)	d(D···A)	<(DHA)	Symmetry code
O(5)-H(5C)...O(1)	1.97	2.819(6)	172.4	x-1/2,y+1/2,z
O(5)-H(5D)...O(1)z	1.92	2.768(6)	172.4	-x+1/2,-y+3/2,-z
O(5)-H(5D)...S(1)	2.96	3.771(4)	160.9	-x+1/2,-y+3/2,-z
O(6)-H(6C)...O(10)	1.81	2.575(11)	148.6	x,-y+1,z-1/2
O(6)-H(6D)...O(13)	1.68	2.47(2)	155.3	-x+1/2,-y+1/2,-z+1
O(7)-H(7C)...O(2)	1.98	2.792(14)	160.5	-x+1/2,-y+1/2,-z+1
O(7)-H(7D)...O(13)	1.19	2.03(3)	170.2	
O(8)-H(8C)...O(3)	1.78	2.621(8)	172.9	
O(8)-H(8C)...S(1)	2.95	3.719(3)	151.5	
O(9)-H(9C)...O(2)	1.91	2.742(7)	167.3	-x+1/2,-y+1/2,-z
O(9)-H(9D)...O(5)	2.07	2.910(7)	167.8	-x,-y+1,-z
O(10)-H(10D)...O(11)	1.97	2.815(11)	174.9	
O(11)-H(11A)...O(13)	2.19	2.89(3)	139.3	x,-y,z-1/2
O(11)-H(11C)...O(5)	2.04	2.888(8)	172.5	x,-y+1,z+1/2
O(12)-H(12C)...O(6)	1.88	2.728(10)	179.2	-x+1/2,-y+1/2,-z+1
O(12)-H(12D)...O(9)	1.92	2.767(7)	177.8	-x+1/2,-y+1/2,-z+1
O(13)-H(13C)...O(4)	1.81	2.65(2)	172.1	-x+1/2,-y+1/2,-z+1
O(13)-H(13C)...S(1)	2.83	3.58(2)	148.1	-x+1/2,-y+1/2,-z+1
Complex 2				
O(7)-H(7A)...O(1)	1.92	2.771(8)	174.3	
O(7)-H(7B)...O(10)	1.90	2.746(14)	173.1	x-1,y,z
O(8)-H(8A)...O(3)	1.86	2.692(10)	164.1	x+1,y-1,z
O(8)-H(8B)...O(15)	1.94	2.766(14)	165.2	
O(9)-H(9A)...O(14)	1.95	2.802(11)	176.5	x+1/2,y-1/2,z
O(9)-H(9B)...O(12)	1.97	2.767(11)	156.7	x+1/2,y-1/2,z
O(10)-H(10A)...O(6)	2.32	3.127(14)	158.6	x+1,y,z
O(10)-H(10B)...O(9)	2.14	2.951(18)	158.2	
O(11)-H(11A)...O(19)	1.87	2.716(18)	177.4	x+1/2,y-1/2,z
O(11)-H(11B)...O(6)	1.93	2.776(10)	177.8	x+1,y,z
O(12)-H(12B)...O(3)	1.88	2.715(10)	169.0	
O(12)-H(12A)...O(13)	2.30	3.093(15)	154.8	
O(13)-H(13A)...O(19)	1.87	2.71(2)	174.0	
O(14)-H(14A)...O(15)	2.06	2.906(12)	178.6	x,y+1,z
O(14)-H(14B)...O(21)	1.95	2.802(12)	179.0	x+1/2,y+1/2,z+1
O(15)-H(15A)...O(4)	1.93	2.775(8)	176.1	x+1,y-1,z
O(15)-H(15B)...O(7)	1.88	2.726(10)	176.6	x+1/2,y-1/2,z
O(16)-H(16A)...O(4)	1.93	2.762(9)	166.4	x+1/2,y-1/2,z
O(16)-H(16B)...O(18)	1.90	2.730(14)	165.1	
O(17)-H(17A)...O(16)	2.13	2.978(12)	173.5	-x+2,y,-z+3/2
O(17)-H(17B)...O(13)	2.14	2.982(13)	173.3	-x+1,y,-z+3/2
O(18)-H(18A)...O(19)	2.33	3.15(2)	163.0	
O(19)-H(19A)...O(18)	2.61	3.15(2)	122.2	
O(20)-H(20B)...O(8)	2.22	3.067(16)	177.2	x-1/2,y+1/2,z-1
O(20)-H(20A)...O(8)	1.74	2.586(15)	173.3	-x+3/2,-y+1/2,-z+1
O(21)-H(21A)...O(9)	2.15	2.992(12)	170.9	-x+1,-y+1,-z+1
O(21)-H(21B)...O(20)	1.91	2.750(14)	170.3	

Symmetry transformations used to generate equivalent atoms

## 3. Experimental

### 3.1. Materials and Physical Measurements

All chemicals were commercial materials of analytical grade and used without further purification. FT-IR spectra were recorded on a Nicolet Magna-IR 550 spectrometer in dry KBr pellets. C, H and N analysis was measured on a MOD 1106 elemental analyzer. The PXRD data were collected on a Bruker D8 diffractometer with Cu Kα radiation (λ = 1.5418Å).

Complexes **1** and **2** were sealed in a capillary with mother liquid for diffraction measurement on a Bruker Smart Apex CCD diffractometer with graphite monochromated Mo Kα radiation (λ = 0.71073 Å ) at 293K for **1** and 296K for 2. All intensity data were corrected for Lorentz and polarization effects (SAINT), and empirical absorption corrections based on equivalent reflections were applied (SADABS). The structure was solved by direct methods and refined by the full-matrix least-squares method on F^2^ with SHELXTL program package. All non-hydrogen atoms of the framework were refined with anisotropic displacement parameters. The organic hydrogen atoms were placed in calculated positions with isotropic displacement parameters set to 1.2×U_eq_ of the attached atom. 

### 3.2. Synthesis of 1-2

#### 3.2.1. Synthesis of [Co_2_(2,2′-bpy)_6_]·(SO_4_)_2_·17H_2_O (**1**)

A solution of 2,2′-bipyridine (156 mg, 1 mmol) in DMF/water (15mL, v/v, 1:1) is added to a solution of CoSO_4_ (155 mg, 1 mmol) in distilled water (10 mL). The mixture is stirred at room temperature for fifteen minutes and placed in a 25 mL Teflon-lined autoclave and heated at 130 °C for 120 h. The autoclave was cooled over a period of 11 h at 10 °C h^-1^, and the product was collected by filtration and dried at ambient temperature to give 1 as pink crystals. Yield: 68%. Anal. Calc. C, 46.39; H, 5.32; N, 10.82%. Found: C, 44.35; H, 5.29; N, 11.08%. FT-IR (cm^-1^): 3,100-3,500 (vs), 1,696 (vs), 1,628 (ms), 1,556 (ms), 1,441 (ms), 1,413 (ms), 1,213 (s), 1,065 (w).

#### 3.2.2. Synthesis of [Cu_2_(2,2′-bpy)_4_(BTCA)]·(OH)(2,2′-bpy)_0.5_14H_2_O (**2**)

A solution of benzene-1,3,5-tricarboxylic acid (210 mg, 1 mmol) in DMF/water (10 mL, v/v, 1:1) is added to the solution of CuCl_2_ (133 mg, 1 mmol) in distilled water (10 mL). Ten minutes later, a solution of 2,2′-bipyridine (156 mg, 1 mmol) in DMF (5mL) is added. The mixture is stirred at room temperature for fifteen minutes and placed in a 25 mL Teflon-lined autoclave and heated at 130 °C for 120 h. The autoclave was cooled over a period of 11h at 10 °C h^-1^, and the product was collected by filtration and dried at ambient temperature to give 2 as pink crystals. Yield: 50%. Anal. Anal. Calc. C, 57.84; H, 4.38; N, 11.18%. Found: C, 57.55; H, 4.39; N, 11.18%. FT-IR (cm^-1^): 3,000-3,500 (vs), 1668 (vs), 1,590 (ms), 1,410 (ms), 1,223 (ms), 1,165 (w), 810 (w).

### 3.3. Single-crystal Structure Determination

Intensity data for **1** and **2** were collected at 296 K on a Bruker SMART CCD area detector diffractometer using graphite-monochromated Mo-Kα radiation (λ = 0.71073 Å) using the *ω-θ* scan mode in the range 0.97 ≤ θ ≤ 25.01°. Raw frame data were integrated with the SAINT [39] program. The structure was solved by direct methods using SHELXS-97 and refined by full-matrix least-squares on *F^2^* using SHELXS-97. An empirical absorption correction was applied with SADABS [40]. All non-hydrogen atoms were refined anisotropically. Hydrogen atoms were set in calculated positions and refined by a riding mode, with a common thermal parameter. All calculations and graphics were performed with SHELXTL and DIAMOND. The crystallographic data and experimental details for the structure analysis are summarized in Table 1.

## 4. Conclusions

In conclusion, we have prepared and characterized two complexes [Co(2,2′-bpy)_3_]·(SO_4_)·8.5H_2_O (1) and [Cu_2_(BTCA) (2,2′-bpy)_4_] (OH)·(2,2′-bpy)_0.5_·14H_2_O (2). Hydrogen bonding interactions and unconventional interactions contribute to the formation of the packing structures. In these structures, 3D hydrogen bonds networks with negative charge are observed. New water morphologies varying from 2D to 3D character have been observed by changing the hydrophobic property of the ligands.

## Supplementary Materials

Supplementary Materials can be seen at http://www.mdpi.com/1420-3049/16/2/2871/s1.
