# Supplementary material for: Cyclic Water Clusters in Tape-Like and Cage-Like Structures
Source: Molecules. 2011 Apr 4;16(4):2871–83. doi: 10.3390/molecules16042871 (PMC6260625; doi:10.3390/molecules16042871)

checkCIF/PLATON report

Structure factors have been supplied for datablock(s) I

No syntax errors found. **CIF dictionary** **Interpreting this report**

Datablock: I

Bond precision: C-C = 0.0074 A Wavelength=0.71073

**Cell:**	**a=22.979(4)**	**b=13.656(3)**	**c=24.890(6)**
	**alpha=90**	**beta=115.235(2)**	**gamma=90**
**Temperature:**	**293 K**		

	Calculated	Reported
Volume	7065(3)	7065(3)
Space group	C 2/c	C2/c
Hall group	-C 2yc	?
Moiety formula	2(C30 H24 Co N6), 2(O4 S), H6 O3, 12(H2 O), H O	?
Sum formula	C60 H79 Co2 N12 O24 S2	C60 H79 Co2 N12 O24 S2
Mr	1534.35	1534.33
Dx,g cm-3	1.442	1.442
Z	4	4
Mu (mm-1)	0.614	0.614
F000	3204.0	3204.0
F000'	3209.30	
h,k,lmax	27,16,29	27,16,29
Nref	6229	6202
Tmin,Tmax	0.874,0.884	0.877,0.887
Tmin'	0.874	

Correction method= MULTI-SCAN

Data completeness= 0.996 Theta(max)= 25.010

R(reflections)= 0.0607(5110) wR2(reflections)= 0.1886(6202)

S = 1.012 Npar= 457

The following ALERTS were generated. Each ALERT has the format
test-name_ALERT_alert-type_alert-level.
Click on the hyperlinks for more details of the test.

Alert level B

```
PLAT417_ALERT_2_B Short Inter D-H..H-D       H5C    ..  H9D     ..        2.09 Ang.
PLAT417_ALERT_2_B Short Inter D-H..H-D       H6C    ..  H10D    ..        2.08 Ang.
PLAT417_ALERT_2_B Short Inter D-H..H-D       H11A   ..  H13C    ..        1.90 Ang.
```

```
PLAT420_ALERT_2_B D-H Without Acceptor       O10    -   H10A   ...          ?
```

Alert level C

```
PLAT232_ALERT_2_C Hirshfeld Test Diff (M-X)  Co1    --  N1      ..       7.72 su
PLAT232_ALERT_2_C Hirshfeld Test Diff (M-X)  Co1    --  N3      ..       6.86 su
PLAT232_ALERT_2_C Hirshfeld Test Diff (M-X)  Co1    --  N4      ..       6.98 su
PLAT232_ALERT_2_C Hirshfeld Test Diff (M-X)  Co1    --  N5      ..       6.11 su
PLAT232_ALERT_2_C Hirshfeld Test Diff (M-X)  Co1    --  N6      ..       6.46 su
PLAT242_ALERT_2_C Check Low       Ueq as Compared to Neighbors for        Co1
PLAT303_ALERT_2_C Full Occupancy H-Atom  H7D    with # Connections          2
PLAT341_ALERT_3_C Low Bond Precision on  C-C Bonds (x 1000) Ang ..          7
PLAT417_ALERT_2_C Short Inter D-H..H-D       H5D    ..  H11C    ..       2.13 Ang.
PLAT911_ALERT_3_C Missing # FCF Refl Between THmin & STh/L=  0.595         27
PLAT918_ALERT_3_C Reflection(s) # with I(obs) much smaller I(calc)          1
PLAT048_ALERT_1_C MoietyFormula Not Given ........................          ?
PLAT125_ALERT_4_C No '_symmetry_space_group_name_Hall' Given .....          ?
PLAT244_ALERT_4_C Low   'Solvent' Ueq as Compared to Neighbors of          S1
```

Alert level G

```
PLAT072_ALERT_2_G SHELXL First  Parameter in WGHT Unusually Large.       0.11
PLAT083_ALERT_2_G SHELXL Second Parameter in WGHT Unusually Large.      19.58
PLAT199_ALERT_1_G Check the Reported _cell_measurement_temperature        293 K
PLAT200_ALERT_1_G Check the Reported   _diffrn_ambient_temperature        293 K
PLAT302_ALERT_4_G Note: Anion/Solvent Disorder ...................       4.00 Perc.
```

```
  0 ALERT level A = In general: serious problem
  4 ALERT level B = Potentially serious problem
 14 ALERT level C = Check and explain
  5 ALERT level G = General alerts; check

  3 ALERT type 1 CIF construction/syntax error, inconsistent or missing data
 14 ALERT type 2 Indicator that the structure model may be wrong or deficient
  3 ALERT type 3 Indicator that the structure quality may be low
  3 ALERT type 4 Improvement, methodology, query or suggestion
  0 ALERT type 5 Informative message, check
```

checkCIF publication errors

Alert level A

```
PUBL004_ALERT_1_A  The contact author's name and address are missing,
            _publ_contact_author_name and _publ_contact_author_address.
PUBL005_ALERT_1_A  _publ_contact_author_email, _publ_contact_author_fax and
            _publ_contact_author_phone are all missing.
            At least one of these should be present.
PUBL006_ALERT_1_A  _publ_requested_journal is missing
            e.g. 'Acta Crystallographica Section C'
PUBL008_ALERT_1_A  _publ_section_title is missing. Title of paper.
PUBL009_ALERT_1_A  _publ_author_name is missing. List of author(s) name(s).
PUBL010_ALERT_1_A  _publ_author_address is missing. Author(s) address(es).
PUBL012_ALERT_1_A  _publ_section_abstract is missing.
            Abstract of paper in English.
```

Alert level G

```
PUBL013_ALERT_1_G The _publ_section_comment (discussion of study) is
            missing. This is required for a full paper submission (but is
            optional for an electronic paper).
PUBL017_ALERT_1_G The _publ_section_references section is missing or
            empty.
```

---

   7 **ALERT level A** = Data missing that is essential or data in wrong format
   2 **ALERT level G** = General alerts. Data that may be required is missing

---

Publication of your CIF

You should always attempt to resolve as many as possible of the alerts in all categories. Often the minor alerts point to easily fixed oversights, errors and omissions in your CIF or refinement strategy, so attention to these fine details can be worthwhile. In order to resolve some of the more serious problems it may be necessary to carry out additional measurements or structure refinements. However, the nature of your study may justify the reported deviations from the submission requirements of the journal and these should be commented upon in the discussion or experimental section of a paper - after all, they might represent an interesting feature.

If level A alerts remain, which you believe to be justified deviations, and you intend to submit this CIF for publication in Acta Crystallographica Section C or Section E, you should additionally insert an explanation in your CIF using the Validation Reply Form (VRF) below. Your explanation will be considered as part of the review process.

If you intend to submit to another section of Acta Crystallographica or Journal of Applied Crystallography or Journal of Synchrotron Radiation, you should make sure that at least a basic structural check is run on the final version of your CIF prior to submission.

```
# start Validation Reply Form
_vrf_PUBL004_GLOBAL
;
PROBLEM: The contact author’s name and address are missing,
RESPONSE: ...
;
_vrf_PUBL005_GLOBAL
;
PROBLEM: _publ_contact_author_email, _publ_contact_author_fax and
RESPONSE: ...
;
_vrf_PUBL006_GLOBAL
;
PROBLEM: _publ_requested_journal is missing
RESPONSE: ...
;
_vrf_PUBL008_GLOBAL
;
PROBLEM: _publ_section_title is missing. Title of paper.
RESPONSE: ...
;
_vrf_PUBL009_GLOBAL
;
PROBLEM: _publ_author_name is missing. List of author(s) name(s).
RESPONSE: ...
;
```

```
_vrf_PUBL010_GLOBAL
;
PROBLEM: _publ_author_address is missing. Author(s) address(es).
RESPONSE: ...
;
_vrf_PUBL012_GLOBAL
;
PROBLEM: _publ_section_abstract is missing.
RESPONSE: ...
;
# end Validation Reply Form
```

If you wish to submit your CIF for publication in Acta Crystallographica Section C or E, you should upload your CIF via the web. If your CIF is to form part of a submission to another IUCr journal, you will be asked, either during electronic submission or by the Co-editor handling your paper, to upload your CIF via our web site.

PLATON version of 22/10/2010; check.def file version of 11/10/2010

Datablock I - ellipsoid plot

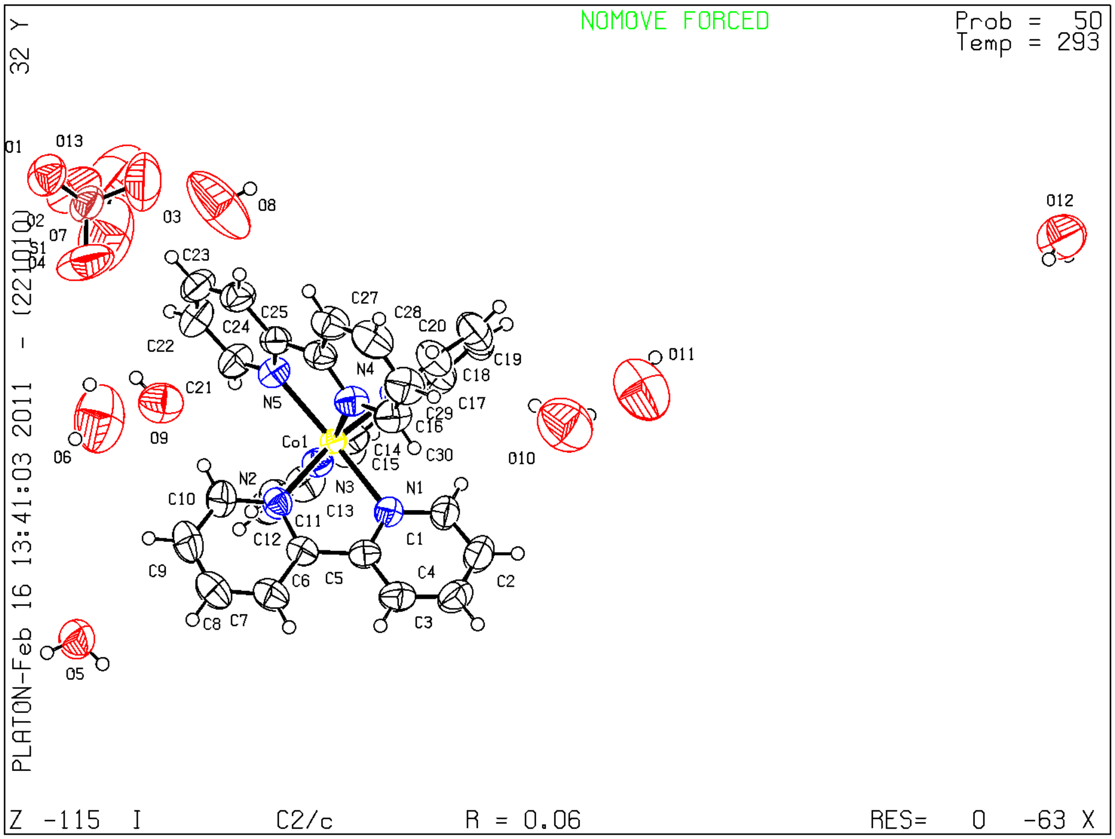

checkCIF/PLATON report

Structure factors have been supplied for datablock(s) I

No syntax errors found. **CIF dictionary** **Interpreting this report**

Datablock: I

Bond precision: C-C = 0.0112 A Wavelength=0.71073

**Cell:**	**a=12.551(5)**	**b=23.483(9)**	**c=42.330(17)**
	**alpha=90**	**beta=95.053(6)**	**gamma=90**
**Temperature:**	**296 K**		

	Calculated	Reported
Volume	12428(8)	12428(9)
Space group	C 2/c	C2/c
Hall group	-C 2yc	?
Moiety formula	C49 H35 Cu2 N8 O6, 0.5(C10 H8 N2), 15(H2 O)	?
Sum formula	C54 H69 Cu2 N9 O21	C54 H69 Cu2 N9 O21
Mr	1307.28	1307.26
Dx,g cm-3	1.397	1.397
Z	8	8
Mu (mm-1)	0.765	0.765
F000	5456.0	5456.0
F000'	5463.72	
h,k,lmax	14,27,50	14,27,50
Nref	10950	10937
Tmin,Tmax	0.777,0.795	0.786,0.803
Tmin'	0.777	

Correction method= MULTI-SCAN

Data completeness= 0.999 Theta(max)= 25.010

R(reflections)= 0.0821(7147) wR2(reflections)= 0.2538(10937)

S = 1.027 Npar= 775

The following ALERTS were generated. Each ALERT has the format
test-name_ALERT_alert-type_alert-level.
Click on the hyperlinks for more details of the test.

Alert level B

```
PLAT415_ALERT_2_B Short Inter D-H..H-X       H13B   ..  H38     ..        2.07 Ang.
PLAT415_ALERT_2_B Short Inter D-H..H-X       H18B   ..  H40     ..        2.04 Ang.
PLAT417_ALERT_2_B Short Inter D-H..H-D       H8B    ..  H15A    ..        2.07 Ang.
```

```
PLAT417_ALERT_2_B Short Inter D-H..H-D       H18A   ..  H19A    ..       1.88 Ang.
PLAT417_ALERT_2_B Short Inter D-H..H-D       H18A   ..  H19B    ..       2.05 Ang.
PLAT420_ALERT_2_B D-H Without Acceptor       O13    -   H13B   ...         ?
PLAT420_ALERT_2_B D-H Without Acceptor       O18    -   H18B   ...         ?
PLAT420_ALERT_2_B D-H Without Acceptor       O19    -   H19A   ...         ?
PLAT420_ALERT_2_B D-H Without Acceptor       O19    -   H19B   ...         ?
```

Alert level C

```
RFACR01_ALERT_3_C  The value of the weighted R factor is > 0.25
            Weighted R factor given   0.254
PLAT241_ALERT_2_C Check High      Ueq as Compared to Neighbors for        N9
PLAT242_ALERT_2_C Check Low       Ueq as Compared to Neighbors for       C50
PLAT341_ALERT_3_C Low Bond Precision on  C-C Bonds (x 1000) Ang ..        11
PLAT911_ALERT_3_C Missing # FCF Refl Between THmin & STh/L=  0.595        13
PLAT918_ALERT_3_C Reflection(s) # with I(obs) much smaller I(calc)         1
PLAT048_ALERT_1_C MoietyFormula Not Given ........................         ?
PLAT125_ALERT_4_C No '_symmetry_space_group_name_Hall' Given .....         ?
PLAT234_ALERT_4_C Large Hirshfeld Difference N9     --  C50     ..       0.18 Ang.
```

Alert level G

```
PLAT072_ALERT_2_G SHELXL First  Parameter in WGHT Unusually Large.       0.12
PLAT083_ALERT_2_G SHELXL Second Parameter in WGHT Unusually Large.      58.87
PLAT710_ALERT_4_G Delete 1-2-3 or 2-3-4 Linear Torsion Angle ... #         9
            N3  -CU2 -N2  -C10   -99.00  4.00   1.555   1.555   1.555   1.555
PLAT710_ALERT_4_G Delete 1-2-3 or 2-3-4 Linear Torsion Angle ... #        13
            N3  -CU2 -N2  -C6     82.00  4.00   1.555   1.555   1.555   1.555
PLAT710_ALERT_4_G Delete 1-2-3 or 2-3-4 Linear Torsion Angle ... #        17
            N2  -CU2 -N3  -C11  -141.00  4.00   1.555   1.555   1.555   1.555
PLAT710_ALERT_4_G Delete 1-2-3 or 2-3-4 Linear Torsion Angle ... #        21
            N2  -CU2 -N3  -C15    39.00  4.00   1.555   1.555   1.555   1.555
PLAT710_ALERT_4_G Delete 1-2-3 or 2-3-4 Linear Torsion Angle ... #        33
            N7  -CU1 -N5  -C25   -14.00  5.00   1.555   1.555   1.555   1.555
PLAT710_ALERT_4_G Delete 1-2-3 or 2-3-4 Linear Torsion Angle ... #        37
            N7  -CU1 -N5  -C21   170.00  4.00   1.555   1.555   1.555   1.555
PLAT710_ALERT_4_G Delete 1-2-3 or 2-3-4 Linear Torsion Angle ... #        49
            N5  -CU1 -N7  -C35  -112.00  5.00   1.555   1.555   1.555   1.555
PLAT710_ALERT_4_G Delete 1-2-3 or 2-3-4 Linear Torsion Angle ... #        53
            N5  -CU1 -N7  -C31    67.00  5.00   1.555   1.555   1.555   1.555
PLAT794_ALERT_5_G Note: Tentative Bond Valency for Cu1     .......       2.31
PLAT794_ALERT_5_G Note: Tentative Bond Valency for Cu2     .......       2.30
```

---

   0 **ALERT level A** = In general: serious problem
   9 **ALERT level B** = Potentially serious problem
   9 **ALERT level C** = Check and explain
  12 **ALERT level G** = General alerts; check

```
   1 ALERT type 1 CIF construction/syntax error, inconsistent or missing data
  13 ALERT type 2 Indicator that the structure model may be wrong or deficient
   4 ALERT type 3 Indicator that the structure quality may be low
  10 ALERT type 4 Improvement, methodology, query or suggestion
   2 ALERT type 5 Informative message, check
```

---

checkCIF publication errors

Alert level A

```
PUBL004_ALERT_1_A  The contact author's name and address are missing,
            _publ_contact_author_name and _publ_contact_author_address.
PUBL005_ALERT_1_A  _publ_contact_author_email, _publ_contact_author_fax and
            _publ_contact_author_phone are all missing.
            At least one of these should be present.
PUBL006_ALERT_1_A  _publ_requested_journal is missing
            e.g. 'Acta Crystallographica Section C'
PUBL008_ALERT_1_A  _publ_section_title is missing. Title of paper.
PUBL009_ALERT_1_A  _publ_author_name is missing. List of author(s) name(s).
PUBL010_ALERT_1_A  _publ_author_address is missing. Author(s) address(es).
PUBL012_ALERT_1_A  _publ_section_abstract is missing.
            Abstract of paper in English.
```

Alert level G

```
PUBL013_ALERT_1_G The _publ_section_comment (discussion of study) is
            missing. This is required for a full paper submission (but is
            optional for an electronic paper).
PUBL017_ALERT_1_G The _publ_section_references section is missing or
            empty.
```

7 **ALERT level A** = Data missing that is essential or data in wrong format
2 **ALERT level G** = General alerts. Data that may be required is missing

Publication of your CIF

You should always attempt to resolve as many as possible of the alerts in all categories. Often the minor alerts point to easily fixed oversights, errors and omissions in your CIF or refinement strategy, so attention to these fine details can be worthwhile. In order to resolve some of the more serious problems it may be necessary to carry out additional measurements or structure refinements. However, the nature of your study may justify the reported deviations from the submission requirements of the journal and these should be commented upon in the discussion or experimental section of a paper - after all, they might represent an interesting feature.

If level A alerts remain, which you believe to be justified deviations, and you intend to submit this CIF for publication in Acta Crystallographica Section C or Section E, you should additionally insert an explanation in your CIF using the Validation Reply Form (VRF) below. Your explanation will be considered as part of the review process.

If you intend to submit to another section of Acta Crystallographica or Journal of Applied Crystallography or Journal of Synchrotron Radiation, you should make sure that at least a basic structural check is run on the final version of your CIF prior to submission.

```
# start Validation Reply Form
_vrf_PUBL004_GLOBAL
;
PROBLEM: The contact author's name and address are missing,
RESPONSE: ...
;
_vrf_PUBL005_GLOBAL
;
```

```
PROBLEM: _publ_contact_author_email, _publ_contact_author_fax and
RESPONSE: ...
;
_vrf_PUBL006_GLOBAL
;
PROBLEM: _publ_requested_journal is missing
RESPONSE: ...
;
_vrf_PUBL008_GLOBAL
;
PROBLEM: _publ_section_title is missing. Title of paper.
RESPONSE: ...
;
_vrf_PUBL009_GLOBAL
;
PROBLEM: _publ_author_name is missing. List of author(s) name(s).
RESPONSE: ...
;
_vrf_PUBL010_GLOBAL
;
PROBLEM: _publ_author_address is missing. Author(s) address(es).
RESPONSE: ...
;
_vrf_PUBL012_GLOBAL
;
PROBLEM: _publ_section_abstract is missing.
RESPONSE: ...
;
# end Validation Reply Form
```

If you wish to submit your CIF for publication in Acta Crystallographica Section C or E, you should upload your CIF via the web. If your CIF is to form part of a submission to another IUCr journal, you will be asked, either during electronic submission or by the Co-editor handling your paper, to upload your CIF via our web site.

PLATON version of 22/10/2010; check.def file version of 11/10/2010

Datablock I - ellipsoid plot

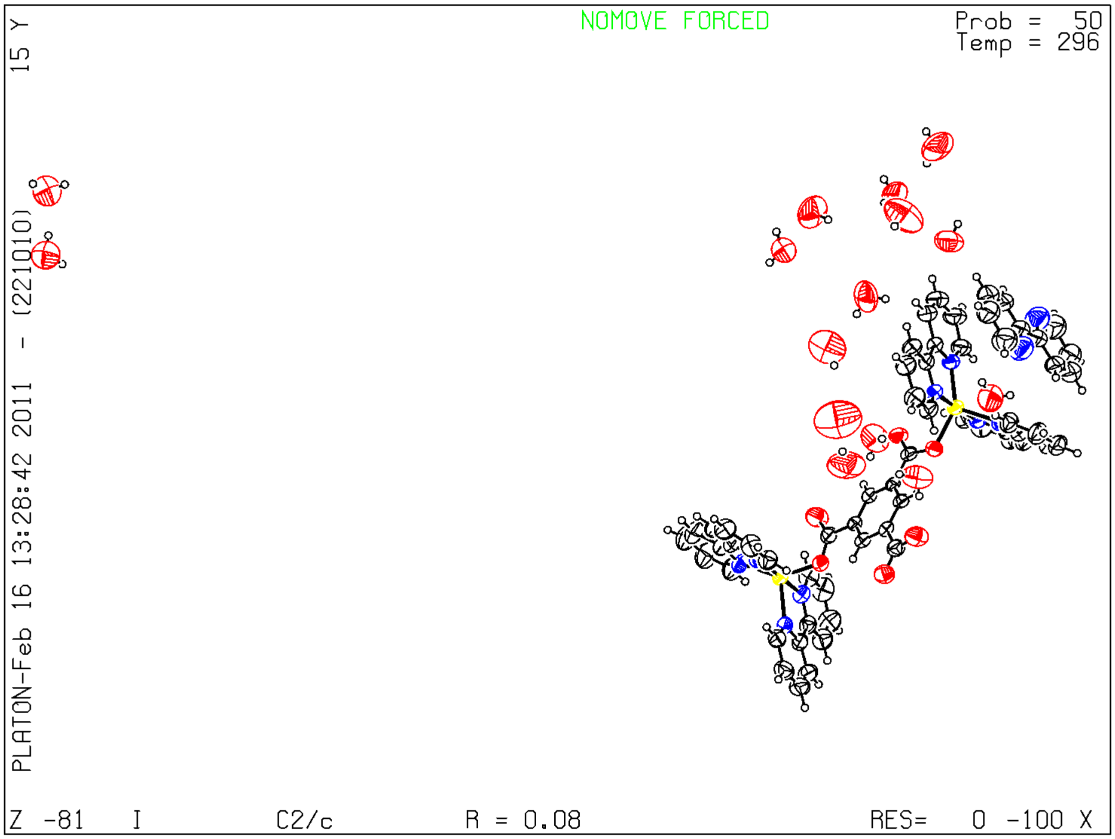

Supplement: Supplementary file 1 [file molecules-16-02871-s001.pdf]
